# A natural goldmine of binding proteins and soluble receptors simplified their translation to blockbuster drugs, all in one decade

**DOI:** 10.3389/fimmu.2023.1151620

**Published:** 2023-02-16

**Authors:** Daniela Novick

**Affiliations:** Molecular Genetics, The Weizmann Institute of Science, Rehovot, Israel

**Keywords:** ligand affinity chromatography, TBPII, IFNAR2, IL-18BP, Enbrel®, Rebif®, Tadekinig alfa ^TM^, COVID 19

## Abstract

Human urinary proteins are a goldmine of natural proteins a feature that simplifies their translation to biologics. Combining this goldmine together with the ligand-affinity-chromatography (LAC) purification method, proved a winning formula in their isolation. LAC specificity, efficiency, simplicity and inherent indispensability in the search for predictable and unpredictable proteins, is superior to other separation techniques. Unlimited amounts of recombinant cytokines and monoclonal antibodies (mAb) accelerated the “triumph”. My approach concluded 35 years of worldwide pursuit for Type I IFN receptor (IFNAR2) and advanced the understanding of the signal transduction of this Type of IFN. TNF, IFNγ and IL-6 as baits enabled the isolation of their corresponding soluble receptors and N-terminal amino acid sequence of the isolated proteins facilitated the cloning of their cell surface counterparts. IL-18, IL-32, and heparanase as the baits yielded the corresponding unpredictable proteins: the antidote IL-18 Binding Protein (IL-18BP), the enzyme Proteinase 3 (PR3) and the hormone Resistin. IFNβ proved beneficial in Multiple Sclerosis and is a blockbuster drug, Rebif^®^. TNF mAbs translated into Remicade^®^ to treat Crohn’s disease. Enbrel^®^ based on TBPII is for Rheumatoid Arthritis. Both are blockbusters. Tadekinig alfa™, a recombinant IL-18BP, is in phase III clinical study for inflammatory and autoimmune diseases. Seven years of continuous compassionate use of Tadekinig alfa™ in children born with mutations (NLRC4, XIAP) proved life-saving and is an example of tailored made medicine. IL-18 is a checkpoint biomarker in cancer and IL-18BP is planned recently to target cytokine storms resulting from CAR-T treatment and in COVID 19.

## 1 Introduction

Soluble cytokine receptors and binding proteins are present in normal body fluids is the concept I established in 1989 once I isolated four such receptors in one month: TNF Binding Proteins I and II (TBPI and TBPII), interferon gamma receptor (IFNγR) and IL-6 receptor (IL-6R) (1, 2). The others, interferon alpha/beta receptor (later named IFNAR2) (3), a soluble fragment of LDL receptor (LDLR) (4), IL-18 Binding Protein (IL-18BP) (5), IL-32 Binding Protein, the enzyme Proteinase 3 (PR3) (6) and Heparanse Binding Protein, the hormone Resistin (7), quickly followed. My approach involved the use of a body fluid, the urine, reflecting naturally occurring proteins, and a highly specific and efficient separation method, ligand affinity chromatography (8). Urine and plasma contain similar repertoire of proteins yet urine has an advantage over plasma since it hardly contains albumin and immunoglobulins, that are too big to be filtered by the kidney, thus stands a concentration of 1000-fold, which is crucial when targeting proteins present in traces amount. Moreover, employing ligand affinity chromatography as the separation method rather than methods based on the chemistry of the target proteins, promises not only the isolation of all binding proteins to a given ligand but also points to its biological activity since it binds the ligand through its active site and also preserves its bioactivity. Yet strategy does not suffice to guarantee success. High amounts of the relevant cytokines and the corresponding monoclonal antibodies are required for purification, monitoring and characterization of the products and we worked out these tools prior to the endeavor of isolation of binding proteins (9). We developed concomitantly the corresponding ELISAs thus we could establish worldwide collaborations to measure the levels of the cytokines and their binding proteins in real life, namely, in health and disease. Moreover, we introduced the concept of free cytokine levels in pathology and calculated it. Free cytokine results from an imbalance of the players and is most probably responsible for the pathology (10, 11). Drug companies recognized the therapeutic potential of the various soluble receptors and binding proteins and translated some into drugs.

## 2 The discovery of cytokine receptors and binding proteins

In 1987, encouraged by the isolation of the EGF receptor *via* affinity chromatography (12), I purified the IFNγR. The protein source was a detergent-extract of huge batches of primary cells (10^11^ foreskin fibroblasts/batch) and the separation method was ligand affinity chromatography, namely, a column of an in-house recombinant IFNγ covalently coupled to a resin (13). Based on this experience I was confident that ligand affinity chromatography would work for other target proteins too provided ligands in milligram amounts are available and so is a rich source of naturally occurring proteins. From now on the source was 500 to 1000-fold concentrated normal human urine.

Soluble receptors are by definition homologous to the extracellular domains of their cell surface counterparts and are thus encoded by the same genes and are generated by a mechanism of alternative splicing or protease cleavage. Binding Proteins deviate from this definition, are not the corresponding receptors and are encoded by separate genes. The advantage of my approach is that it isolates both types of these molecules provided a given ligand has a sufficient affinity to the putative soluble receptor or binding protein.

### 2.1 Soluble TNF receptors (1989) and anti TNF therapy

The ultimate proof that my strategy works beyond expectations was the isolation of TBPI and TBPII. 20,000-fold purification in one step and over 80% recovery of bioactivity were achieved (1). It should be noted that purification *via* the canonical multistep laborious and inefficient chromatographic procedures yielded TBPI only (14). Of particular note is the fact that my approach, namely ligand affinity chromatography of urine on covalently coupled TNF to a resin, yielded not only the TBPI but also the novel at that time TBPII and it was the TBPII that proved beneficial in patients with Rheumatoid Arthritis. TBPI and TBPII were hypothesized to be soluble receptors and indeed based on their amino acid sequence their cell surface counterparts were easily cloned. Discovered in 1975, the TNF cytokine (15) proved with years to be a master cytokine involved in opposing biological activities. Together with its receptors and transcription factors TNF was shown to play a role in cell death and cell survival, to regulate immune functions and to be involved in many pathologies (16). TNF blockers were spotted by drug companies and translated to biologics that soon became blockbuster drugs. The TBPII was translated into Etanercept/Enbrel^®^, a fusion protein with the Fc portion of immunoglobulin, for the treatment of mainly Rheumatoid Arthritis but also other autoimmune and inflammatory pathologies such as Psoriatic arthritis, Juvenile idiopathic arthritis and Ankylosing spondylitis. TNF mAbs were translated into e.g. Remicade^®^ and Humira^®^ for the treatment of Crohn’s disease and Ulcerative Colitis in addition to the pathologies treated by Enbrel^®^. The difference in the mechanism of action between these TNF blockers is addressed by Levin AD et al. (17). In a way, I had been involved in the monoclonal antibody anti TNF therapy too. In 1985 Hahn et al. generated anti TNF mAbs (18). I contributed a unique screening procedure for the selection of these mAbs, a screening developed by our laboratory for the selection of mAbs to various interferons (9). As stated later on, this screening assay was a crucial step in the selection of the TNF mAbs.

The discovery of the TNF receptors lead to a flood of world-wide research on the mechanism of action, signal transduction, immune response, cross talk with other cytokines and involvement in health and disease and this is what placed TNF high in the hierarchy of master cytokines. Biologics that neutralize TNF are amongst the most successful drugs for the treatment of chronic inflammatory and autoimmune pathologies (19, 20). In 1998 a modification of our TBPII and our anti TNF mAbs were the first TNF blockers to be approved by the FDA. I had the privilege to have a major part in their discovery and development.

### 2.2 Soluble receptors to IL-6 and IFNγ (1989)

I had no doubt that in addition to the TBPs urine contains other soluble receptors. Indeed, using different baits, my approach pulled out two additional proteins, the soluble IL-6 receptor (IL-6R) and the soluble Type II interferon receptor (IFNγR). To our surprise, unlike the antagonistic soluble receptors to TNF and to IFNγ, the soluble IL-6R behaved as an agonist (21). In a mechanism named trans-signaling it was shown that it binds its corresponding ligand, the circulating IL-6, and presents it directly to the transducing IL-6R chain, gp130, present on cells that lack the binding IL-6R chain (22). The discovery of this player, the soluble IL-6R, uncovered this additional mode of signaling of IL-6 on top of the IL-6 classical signaling. It also added an important dimension in the development of inhibitors to IL-6 and IL-6R yet additional treatment for Rheumatoid Arthritis and a variety of inflammatory and autoimmune diseases (23, 24).

The type II IFN, IFNγ, named also and immune IFN, is also a master cytokine and is involved in pro-inflammatory and autoimmune pathologies. Obviously, we and others generated monoclonal antibodies to these cytokines and their receptors (9). Anti IFNγ mAbs developed by others translated to Emapalumab^®^, a drug approved in 2018 for primary hemophagocytic lymphohistiocytosis (HLH) (25). In the current pandemic these blocking agents are being considered for the treatment of severe cases of COVID 19.

### 2.3 Soluble IFNα/β Receptor (1992)

Type I interferons were discovered by Isaacs and Lindenmann in 1957 (26) but it took 35 years to uncover their receptor though laboratories all over the world were engaged in seeking it. Once again, my unique approach of isolation came to rescue and my “goldmine” yielded both the desired receptor and an eureka moment for me that I will never forget. Passing an equivalent of 500 Liter of normal human urine on a resin to which IFNα or IFNβ were covalently coupled yielded a few micrograms of a soluble Type I IFN receptor. By definition a soluble receptor is homologous to the extracellular domain of the cell bound receptor, thus the N-terminal amino acid sequence of our soluble receptor served to clone its self-surface counterpart. Two cell bound ligand binding receptors were discovered, ours, named IFNα/β receptor, a decoy receptor with a short intracellular domain (3) and the transducing receptor with the full intracellular length, later named IFNAR2 (27).

The discovery of the Type I IFN receptor was followed by a burst of publications on the mechanism of action of this yet another master cytokine. We showed the stepwise ligand induced formation of the trimeric complex (28) that include the IFN that first binds the ligand binding chain (IFNAR2) and then the chain, discovered earlier by Uze et al., that transduces the signal joins (29). We were the first to demonstrate the physical interaction of the ligand binding receptor with the transcription factor JAK1 (3), and our neutralizing mAbs raised against this receptor pointed to the fact that the JAK-STAT pathway does not explain all tested biological activities of Type I IFN (30).

JAKs have a pivotal role in a variety of immune mediated inflammatory and autoimmune diseases thus their targeted inhibition results in effective disease control (31). The use of JAK blocking drugs is extended in the present pandemic and there are ongoing clinical studies with these agents aiming at attenuation of the over-production of proinflammatory cytokines in severe Covid 19 (32).

Type I IFN was discovered 65 years ago as an antiviral agent. Being a bridge between innate and adaptive immunity, being essential in host defense and involved in cancer and autoimmunity, placed interferon in the limited list of master cytokines. As such it is being continuously revisited and is a source for drug discovery (33).

Forty years after the discovery, one of the Type I IFNs, IFNβ, had been approved for the treatment of multiple sclerosis (34). My persistence and daring paid, and in 1982 I generated monoclonal antibodies to this IFN (9, 35). These tools accelerated the CHO-expressed recombinant IFNβ characterization, monitoring of its scaled-up production, and submission of its file to the FDA. I am proud to have a part in its translation to a blockbuster drug, the REBIF^®^.

Interferons are extensively studied in the present pandemic, the SARS-2 Covid 19. Big data analyses revealed interferon deficiency, inborn errors in IFN signaling and autoantibodies to Type I IFN in severely ill patients. The latter accounts for more than 10% of these patients (36–42).

### 2.4 IL-18 Binding Protein (1997)

Having isolated 5 soluble receptors, gave me confidence that my approach would yield any unknown receptor provided it is present in the goldmine, the urine. But here I experienced a twist to my story. Based on my expertise I had been asked by Prof. Charles Dinarello, known in the field of IL-1, to isolate the receptor for an additional member of the IL-1 family, the pro-inflammatory cytokine, IL-18, first named IFNγ inducing factor (IGIF). To my surprise, all my attempts to isolate the soluble IL-18 receptor from the concentrated urine failed not because it does not exist but because in retrospect it turned out that its concentration in the urine and its affinity to the IL-18 are too low. Characterization of the protein that I did pull out instead, revealed a novel family of proteins, the Binding Proteins, osteoprotegerin being then its only member. These proteins bind the same ligand as the corresponding receptor does, but are encoded by a separate gene and have no cell surface counterpart. The protein I isolated was a unique binding protein, that we named IL-18BP (5). It has an exceptionally high affinity to its ligand (0.4 nM or 0.05 nM) (43, 44) and as such was proposed to serve as an antidote in a fatal inherited IL-18BP deficiency in human fulminant viral hepatitis A caused by toxic levels of IL-18 (45). Serono (Merck) translated recombinant IL-18BP to a drug and named it Tadekinig alfa™. There is an ongoing phase III clinical trial by AB2 bio (https://www.ab2bio.com) in children born with a mutation in the inflammasome e.g. *NLRC4* and in *XIAP/BIRC4* (ClinicalTrials.gov Identifier: NCT03113760). These mutations lead to an over expression of IL-18 that results in organ damage due to Macrophage Activation Syndrome (MAS) or hemophagocytic lymphohistiocytosis (HLH). On a compassionate basis Tadekinig alfa™ saved several children lives and let them lead almost normal life by being treated continuously for 7 years now. A successful phase II clinical study in the autoimmune Still’s disease is completed and awaiting phase III. Tadekinig alfa™ treatment protocol was also submitted to the FDA to be tested in patients succumbing to a devastating MAS, resulting from cancer and viral diseases that otherwise has no cure and has up to 50% mortality. Tadekinig alfa™ is considered in the treatment of a cytokine storm in patients undergoing CAR T therapy (46) and in severe cases of COVID 19 (47). The complete story referring to IL-18BP discovery can be found in my recent review (9).

### 2.5 Soluble LDL receptor, IL-32 and heparanase binding proteins

My approach proved indispensable in the isolation of binding proteins not only to cytokines but also to other key molecules such as LDL and heparanase. We found that a soluble LDL receptor, present in urine, has an unanticipated antiviral activity and that its cell surface counterpart, present in all types of cells, is the entry receptor of VSV (4, 48). These findings explain the pantropism of this virus used successfully in gene therapy.

An attempt to isolate the receptor to the IL-18 induced proinflammatory cytokine, the IL-32, failed and I isolated Proteinase 3 (PR3) instead (6). PR3 is the auto-antigen of an autoimmune blood vessel disease, Wegener’s disease renamed Granulomatosis with polyangiitis (GPA). Blood levels of IL-32 were reported to be upregulated in these patients.

The receptor to heparanase was not isolated either but instead we have shown that the human resistin, present in urine, binds heparanse specifically and with a high affinity (7). Resistin is an adipogen in mice and a pro-inflammatory cytokine in humans (49, 50). The heparanase receptor is unknown till today.

## 3 Concluding remarks

The notion coined back in 1968 by Cuatrecasas et al. (8) that almost any given biomolecule has an inherent recognition site through which it recognizes a partner molecule served as the basis to my approach. My findings upgraded the status of normal human urine to a goldmine position, and the convenience in its handling placed it high in the list of sources of natural proteins such as soluble receptors and binding proteins. No doubt being self-proteins facilitated their translation into drugs.

It is clear now that the balance between the cytokines and their antagonists, agonists, or carrier proteins, namely, the soluble receptors and binding protein, dictate the outcome of a pathology. This is where I introduced the concept of a free cytokine (10, 51).

A spectacular comeback at the present pandemic, SARS-2 Covid 19, of these master cytokines and their receptors reminded all how crucial they are. TNF, IL-6, both types of IFNs and IL-18 were reported to be involved in the cytokine storm of severely ill Covid 19 patients. Blockers of these cytokines are being tested as possible therapeutic agents.

Though the journey taken to generate the tools and to discover the various receptors and binding proteins seems simple and straightforward it was not. The details of the “struggle” that lead to my success where others failed, my discoveries, the rationale behind and the “tricks” engaged, are described in my historical review titled “Nine receptors and binding proteins, four drugs, and one woman: Historical and personal perspectives” (9). I should stress that at those days all our research was basic-science oriented with a desire to solve Nature’s enigmas yet also with a remote dream that part of it will prove beneficial to humanity. Mine did in a form of Rebif^®^, Enbrel^®^, Remicade^®^ and Tadekinig alfa^™^.

## Author contributions

DN is the sole author. She conceived and wrote the manuscript.
